# Axonal Damage in Pediatric Multiple Sclerosis

**DOI:** 10.15844/pedneurbriefs-29-5-1

**Published:** 2015-05

**Authors:** Nancy L. Kuntz

**Affiliations:** 1 Division of Neurology, Ann & Robert H. Lurie Children's Hospital of Chicago, Chicago, IL; 2 Departments of Pediatrics and Neurology, Northwestern University Feinberg School of Medicine, Chicago, IL

**Keywords:** Pediatric, Multiple Sclerosis, Axonal Damage

## Abstract

Investigators from Georg August University, Gottingen, Germany, analyzed axonal pathology in brain biopsy and autopsy samples from 19 children with early multiple sclerosis (MS).

Investigators from Georg August University, Gottingen, Germany, analyzed axonal pathology in brain biopsy and autopsy samples from 19 children with early multiple sclerosis (MS). As compared to adults with MS, children present with polyfocal onset of symptoms, have a higher relapse rate during early years of the disease yet have shorter time to recovery and a higher rate of remission.

Histopathologic features of CNS biopsies (and 1 autopsy specimen) from children with pediatric MS collected over 18 years (1997-2014) at two academic medical centers were assessed and compared to features of CNS tissue from adults with MS. Nineteen of 64 pediatric specimens contained sufficient tissue sample for analysis of axonal damage. Markers of axonal injury included reduction in axonal density and presence of axonal spheroids positive for amyloid precursor protein (APP).

A 49% reduction in axonal density within demyelinating lesions in pediatric biopsies was calculated in comparison to periplaque white matter. Acute axonal damage was increased in early active demyelinating lesions in children as compared to adult biopsies and was negatively correlated with age of the subject at time of biopsy. An increased density of APP-positive axons in pediatric early demyelinating lesions was noted particularly in biopsies of children who were < 11 years of age (pre-pubertal) in whom the counts were 50% higher than those in post-pubertal or adult biopsies. Expanded Disability Status Scale at onset of the attack correlated with the number of APP positive spheroids in the biopsy. The amount of macrophage infiltration correlated with the degree of axonal injury in both pediatric and adult biopsies. [1]

COMMENTARY. The authors point out one of the potential limitations of their report: the selection bias relating to which clinical cases of CNS demyelinating disease were biopsied. In the cases with histology available, an atypical or unusual feature of presentation (clinical, imaging, or severity) had been distinctive enough to warrant biopsy to exclude an alternative diagnosis. Clinical cases in which the clinical, imaging and diagnostic parameters all cleanly fit within an expected range of findings would be diagnosed and treated without biopsy. However, the care with which these authors staged the acuity and phase of demyelinating lesions, their comparison of the axonal density within the lesion to that in the periplaque white matter and the contrast of all of these parameters with adult biopsies from clinically similar events makes these findings independently credible and important.

Other biomarkers have been noted to be different in children (particularly pre-pubertal children) with pediatric MS. CSF has been noted to have more inflammatory cells such as neutrophils and lower likelihood of oligoclonal bands or elevation in IgG index in children [2]. The authors here point out that this likely indicates prominent activation of the innate immune system as compared to the adaptive immune system post puberty. MRI in children with pediatric MS has shown an increased number and size of T2 lesions in the CNS in early disease [3].

Despite the greater degrees of acute axonal damage noted in CNS demyelinating lesions in prepubertal children, their rate of clinical recovery from clinical events is greater than in adults [2]. MR studies support this by noting a greater tendency for demyelinating lesions to “vanish” over time in prepubertal children [4].

This article begins the important process of elucidating the differences in pathophysiology of CNS demyelinating disease in children, particularly young or prepubertal children.
